# A Practical Approach to Red Blood Cell Folate Analysis

**Published:** 2007-11-14

**Authors:** Chandrika J Piyathilake, Constance B Robinson, Phillip Cornwell

**Affiliations:** Department of Nutrition Sciences, University of Alabama at Birmingham, AL 35294

**Keywords:** folate, packed RBC, hemolysate

## Abstract

The measurement of folate in red blood cells (RBCs) is preferred since it reflects long-term folate status in the body compared to plasma/serum folate which may be influenced by recent dietary intake. The commonly accepted technique for RBC folate analysis involves preparation of a hemolysate using a fresh whole blood sample. Hematocrit and plasma folate concentrations are needed to calculate RBC folate values. Because of the need for immediate access to a laboratory where processing can be performed, it may not be practical to assess RBC folate status using this method in field-based epidemiological studies. It is however, feasible to isolate packed RBSs from a blood sample under these conditions. The purpose of this study is to validate RBC folate analysis using packed red cells by comparing the RBC folate values obtained by hemolysate method (routine assay) with those obtained by using packed RBCs (new assay) in the same individuals (n = 50) using the folate microbiological assay. The correlation between plasma folate and the routine RBC folate assay (r = 0.58, p = 0.001) and the correlation between plasma folate and the new RBC folate assay was statistically significant (r = 0.55, p = 0.001). The correlation between RBC folate by the routine assay and new assay was also statistically significant (r = 0.78, p < 0.001). We conclude that measurement of folate in packed RBC is a practical approach in assessing long-term folate status in field-based and or larger scale epidemiological studies where an immediate access to a laboratory is unavailable for necessary sample processing for the routine RBC folate assay.

## Introduction

Circulating blood folate analysis has been the routine diagnostic test for folate deficiency for over three decades. Assessment of folate status has also been important because of its role in reducing the risk for cardiovascular disease [1], neural tube defects [2] and cancers [3]. The measurement of folate in red blood cells (RBCs) is preferred since it reflects long-term folate status in the body compared to plasma/serum folate which may be influenced by recent dietary intake [4]. The commonly accepted technique for RBC folate analysis involves preparation of a hemolysate using fresh whole blood by diluting it in freshly prepared 1% ascorbate. Incubation of the hemolysate at 37 ºC for 20 minutes allows endogenous plasma conjugase (gamma-glutamyl carboxypeptidase) to convert RBC folate polyglutamates to assayable folates. Because of the need for immediate access to a laboratory where hemolysates can be prepared appropriately, it may not be practical to assess RBC folate status in field-based epidemiological studies. It is however, feasible to isolate packed red blood cells from a blood sample under these conditions. The purpose of this study is to validate RBC folate analysis using packed red cells by comparing the RBC folate values obtained by hemolysate method with those obtained by using packed RBCs in the same individuals.

## Materials and Methods

We used 50 randomly selected samples which were processed and stored from a large study where all study participants gave permission to use their samples in future studies related to cancer research. These samples had been collected over a 12-month period. All these samples were immediately processed and stored appropriately to assess plasma and RBC folate by using a RBC hemolysate method. Briefly, a 10 ml blood sample was collected into one EDTA (purple top) vacutainer tube. The hematocrit (needed to calculate RBC folate concentrations) was measured using 25 μl of whole blood. After mixing 25 μl of whole blood with 725 μl of freshly prepared 1% ascorbate for the RBC folate assay, the remainders of the whole blood samples were centrifuged at 3000 rpm for 10 minutes to separate plasma from RBCs. Plasma was transferred to a separate tube and stored at −80 ºC until used for folate analysis. Buffy coat was taken off carefully to remove all white blood cells from the sample. The packed red cells were transferred to a centrifuge tube and stored at −80 ºC until used for future assays. In this study, we used plasma, RBC hemolysate and packed RBCs for folate analysis from the selected individuals.

## Preparation of RBCs for Folate Analysis

When freshly collected blood samples were used for RBC folate assay, the conversion of RBC folate polyglutamates to monoglutamates was achieved enzymatically by plasma folate conjugase after incubating the hemolysate (prepared by mixing 25 μl of whole blood with 725 μl of freshly prepared 1% ascorbic acid) at 37 ºC for 20 minutes. Rat plasma was used as a source of conjugase to convert folate polyglutamates to monoglutamates in packed RBCs. Rat plasma (Harland Bioproducts for Science, Catalog # BT-4511) was treated with activated charcoal (Sigma, Catalog # C-4386)) to remove folate; 750 mg of charcoal per 15 ml of rat plasma was stirred very gently for 60 minutes on ice and centrifuged at 3500 rpm at 4 °C for 5 minutes. The supernatant was filtered through a 0.22 micron filter. After the rat plasma was tested for folate to make sure that it is free of folate, aliquots were made and stored at −70 °C. Initial experiments indicated that optimal conversion of folate polyglutamates in RBC samples can be achieved by mixing 25 μl of packed RBCs with 712.5 μl of freshly prepared 1% ascorbic acid and 12.5 μl of rat plasma (charcoal stripped) and by incubating the mixture at 37 ºC for 30 minutes. All packed red cell samples were prepared following this protocol.

## L. casei Microbiological Assay

The 96-well plate adaptation of L. casei microbiological assay was used to measure the total folate levels in the appropriately treated samples of RBC and plasma [5,6,7]. Briefly, 20 μl of sample and folate standard (15 ng/ml) was used in the assay. The volumes of the sample and of the standard were adjusted to 300 μl using 0.1 M phosphate buffer (pH 8.6) containing 10 mg/ml of ascorbic acid. Six serial dilutions of sample and standard (folate standards in the range of 0.005 to 0.15 ng/0.3 ml assay well after serial dilutions) were made using a 12-channel pipetter. Then 150 μl of L. casei medium containing L. casei, at a concentration of 5 μl/10 ml of medium) was pipetted into all wells and incubated for 18 hours at 37 ºC. After incubation, the contents of each well were suspended by repeated aspiration and flushing several times with a 12-channel pipetter. Bacterial growth was measured by reading the optical density at 655 nm. This assay is based on the ability of the folate in the sample to stimulate Lactobacillus casei (ATTC 7469) growth relative to folate-containing standards. The coefficient of variation and recovery of this assay are 5%–7% and 96%–105% respectively. To monitor the reproducibility of these assays, two pooled samples (low and high) prepared from plasma obtained from the American Red Cross was assayed at least 30 times and the means and standard deviations were determined. These served as the basis for the quality control for the assays (the study sample assays were repeated if the values of the control samples in each run were beyond two standard deviations from the mean). The low and high pools were included in every plate. Plasma, RBC hemolysate and packed RBC preparations from the same individuals were assayed for folate under the same experimental conditions (same plate, same day, etc).

## Calculation of RBC Folate

The following formula was used to calculate RBC folate when the routine assay was used.

$$\frac{\begin{array}{c} \text{Whole}\;\text{blood}\;\text{folate}\;(\text{ng}/\text{ml})-[\text{Plasma}\;\text{folate}\;\text{ng}/\text{ml}\\ \left(1-\text{hematocrit}/100)\right] \end{array}}{\text{Hematocrit}/100}$$

This formula makes a correction for plasma folate present in the whole blood hemolysate and for the proportion of blood that consists of RBC. Since packed red cells are used in the new assay, a correction for plasma folate or hematocrit is not required in the new assay. The rat plasma used in this assay was charcoal stripped and was tested to have no detectable folate.

In addition, the experiment described below was performed to evaluate whether the efficiency of rat plasma conjugase is different from that of the human plasma conjugase. To conduct this experiment, blood samples drawn from six individuals were centrifuged at 3000 rpm/10 minutes. The plasma and buffy coat were removed to isolate RBC. Two aliquots of RBC from each sample were treated with rat plasma or human plasma from each individual as described previously.

An additional experiment was also conducted to determine whether the removal of buffy coat makes a difference in RBC folate values. Blood samples from six individuals drawn in duplicate tubes (5 ml each) were used in these experiments. All tubes were centrifuged at 3000 rpm/10 minutes. Plasma was removed from one tube from each individual leaving buffy coat and packed red cells. From the other set of tubes, plasma and buffy coat was removed leaving only the packed red cells. RBC with or without buffy coat was treated with rat plasma as described previously.

## Statistical Analysis

Descriptive statistics such as mean, median, standard deviation (SD) of the mean, and range were computed to examine the differences between the RBC hemolysate method (routine) and RBC packed cell method (new). Wilcoxon Signed Rank test was employed to determine the statistical significance of the differences between folate results obtained by the routine and new techniques. The differences between folate values due to the use of rat plasma or human plasma or the differences in folate values due to removal or leaving the buffy coat were tested with similar statistical tests. Non-parametric (Spearman) correlation analyses were employed to determine the association between folate results of routine and new techniques

## Results

**Figure 1:**
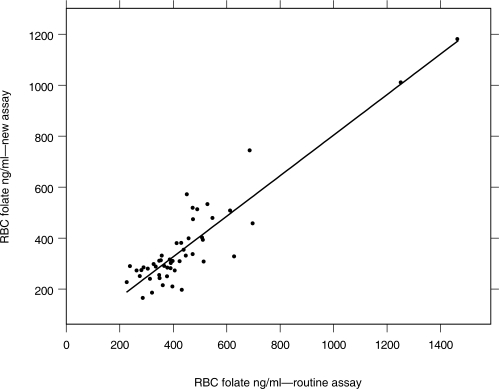
Correlation between RBC folate measured by the routine assay and the new assay.

The mean ± SD and median of RBC folate by the routine assay was 449 ± 216 ng/ml and 399 ng/ml. The mean ± SD and median of RBC folate by the new assay was 366 ± 188 ng/ml and 309 ng/ml. The difference between the two assays was statistically significant (p < 0.001, Wilcoxon Signed Rank Test). In 43 samples, the RBC folate values were higher with the routine assay compared to the new assay and 7 samples had higher RBC folate values with the new assay compared to the routine assay. Over all, with 43 samples, RBC folate values were 23% lower with the new assay compared to the routine assay. With 7 samples, RBC folate values were 10% higher with the new assay compared to the routine assay. The higher or lower RBC values by the new technique compared to the routine technique were distributed unevenly in the data set. There was no inverse association between the lengths of storage of samples over 12 months folate values to indicate that longer storage time may yield lower RBC folate values by the new technique. The correlation between plasma folate and the routine RBC folate assay (r = 0.58, p = 0.001) and the correlation between plasma folate and the new RBC folate assay was statistically significant (r = 0.55, p = 0.001). The correlation between RBC folate by the routine assay and new assay was also statistically significant (r = 0.78, p < 0.001, Figure 1).

On average, the RBC folate values of samples treated with rat plasma were 24% lower compared to the RBC values of samples treated with human plasma and this difference was statistically significant (p = 0.03). RBC folate values of samples with buffy coat had slightly higher folate values compared to RBC samples without buffy coat, but the difference between the two was not statistically significant (p = 0.27).

## Discussion

Folate values can be measured using either a serum/plasma folate or a red cell folate assay. A negative folate balance seen in hospital patients are shown to result in a low serum folate value without folate deficiency [8]. Therefore, low red cell folate, thought to indicate tissue deficiency, may be more important than a low serum folate in the diagnosis of folate deficiency. Longer term folate status is also likely to be more informative in assessing its importance in chronic diseases such as cancerous or pre cancerous conditions. We recently reported that a combined measure of RBC and plasma folate status is a much stronger predictor for the natural history (acquisition, persistence or clearance of high risk human papilloma virus, the main risk factor for CIN and cervical cancer than plasma folate alone [9]. These observations suggest that assessment of RBC folate status is important in field-based epidemiological studies. However, the need for immediate access to a laboratory where hematocrit could be measured and hemolysates can be prepared appropriately is an obstacle for using the routine RBC folate assay in field-based epidemiological studies. For this reason, samples are not available to assess RBC folate status in most currently available biorepositories of epidemiological studies Also, the routine RBC folate calculation requires the value for plasma folate which increases the cost for RBC folate assessment in larger scale studies. RBC folate assessment using packed RBC described in this manuscript eliminates some of these limitations. We also document that RBCs can be stored with or without buffy coats and folate is stable in these samples for a 12-month period. We noted that RBC folate concentrations in a given sample is likely to be lower when rat plasma was used to convert RBC folate polyglutamates to monoglutamates compared to human plasma and this may be due to lower efficiency of rat plasma in converting human RBC polyglutamates to monoglutamates. This should not however, interfere with data interpretation as long as rat plasma is used with all samples in a given study. We have used this newly developed RBC folate assay in a large epidemiological study where we confirmed that the relationship between a polymorphism in the folate pathway and folate concentrations are similar when folate was measured using the new assay and the routine folate assay which used a whole blood hemolysate [10]. In conclusion, we document that measurement of folate in packed RBC is a practical approach in assessing long-term folate status in field-based and or larger scale epidemiological studies.
